# Mitochondrial genome and phylogenetic analysis of *Psylliodes punctifrons* Baly (Coleoptera: Chrysomelidae: Galerucinae)

**DOI:** 10.1080/23802359.2020.1832597

**Published:** 2020-10-27

**Authors:** Li-Hong Wu, Zhi-Yan Wei, Chao-Xing Hu

**Affiliations:** Guizhou Provincial Key Laboratory for Agricultural Pest Management of the Mountainous Region, Scientific Observing and Experimental Station of Crop Pest in Guiyang, Ministry of Agriculture, Institute of Entomology, Guizhou University, Guiyang, P. R. China

**Keywords:** Mitogenome, *Psylliodes punctifrons*, agricultural pest

## Abstract

We sequenced and annotated mitochondrial genome (mitogenome) of oilseed rape pest *Psylliodes punctifrons* Baly for the first time. The mitogenome is 15,611 bp, and the nucleotide composition of 37 genes is highly A + T biased (A: 34.2, C: 11.6, G: 12.1, and T:42.1). All PCGs start with ATN and stop with TAR, except *COX3* and *ND4* that stop with incomplete codon T—. The phylogenetic tree confirms that *P. punctifrons* is clustered with other *Psylliodes* species. This study enriches the mitogenomes of agricultural pests.

The genus of *Psylliodes* Latreille belongs to the subfamily Galerucinae and includes more than 200 species (He et al. 2019). A lot of species within this genus are known as agricultural pests, which can attach plants of some 30 families (Jolivet and Hawkeswood 1995). *Psylliodes punctifrons* is widely distributed throughout China and is pest of all cruciferous vegetables (He et al. 2000). In this study, we have sequenced and determined the mitochondrial genome (mitogenome) of *P. punctifrons* using next-generation sequencing method (Illumina) for the first time. The sequences obtained in this study may facilitate future studies on identification, population genetics, and biological control of *P. punctifrons*.

Total genome DNA of *P. punctifrons* was extracted from male adults that were collected from eggplants, in Guiyang, Guizhou Province, China (106°39′59″E, 26°30′20″N), in June 2020. Total genomic DNA was extracted using DNeasy Blood and Tissue Kit (Qiagen), following the protocol. Paired-end library (450 bp) was sequenced using Illumina Hiseq4000 platform, with 150 bp pair-end sequencing method (Sangon Biotech (Shanghai) Co., Ltd.), after trimming and filtering of the raw sequencing reads, the Q20 bases ratio is 97.00%, generating 24,477,104 high-quality clean reads (7.47 Gb). Remaining samples and genome DNA are deposited in the Institute of Entomology, Guizhou University, Guiyang, China (GUGC).

Mitogenome of *P. punctifrons* was assembled using Geneious primer (v. 2019.2.1) with reference sequences (*Psylliodes hispanus*, KX943503), Assembled mitogenome of *P. punctifrons* is 15,611 bp in length (GenBank No. MT890591), and 39,065 reads were matched to the reference sequences, the mean coverage of the mitogenome is 354.5. Mitogenome of *P. punctifrons* were annotated using MITOS v2 server with the invertebrate genetic codes (Bernt et al. 2013), ARWEN 1.2 (Laslett and Canback 2008) and ORF Finder in NCBI. containing typical 37 mitogenome genes (13 protein-coding genes (PCGs, 11,129 bp), 22 transfer RNA genes (tRNAs, 1,447 bp), and two ribosomal RNA genes (rRNAs, 2021 bp)) and a control region. Generally, the nucleotide composition of 37 genes is highly A + T biased (A: 34.2, C: 11.6, G: 12.1, and T:42.1), except *COX3* and *ND4* stop with incomplete codon T—, all PCGs start with the canonical putative start codon ATN (ATA/ATT/ATC/ATG), and stop with termination codon TAA/TAG. The *16S* rRNA and *12S* rRNA are 1280 bp and 741 bp in length, respectively.

The phylogenetic relationships of *P. punctifrons* were reconstructed with IQ-TREE using an ultrafast bootstrap that approximation approach1000 replicates based on first and second codon positions of the 13 PCGs with 7234 sites. Each PCG sequence was aligned using MAFFT v7.0 and Gblocks 9.1 b (Katoh and Standley 2013) and then concatenated individual genes using MEGAX (Kumar et al. 2018). The phylogenetic tree confirms that *P. punctifrons* is clustered with other *Psylliodes* species with high support (100) (Figure 1). We hope that our data can be useful for further study.

**Figure 1.**
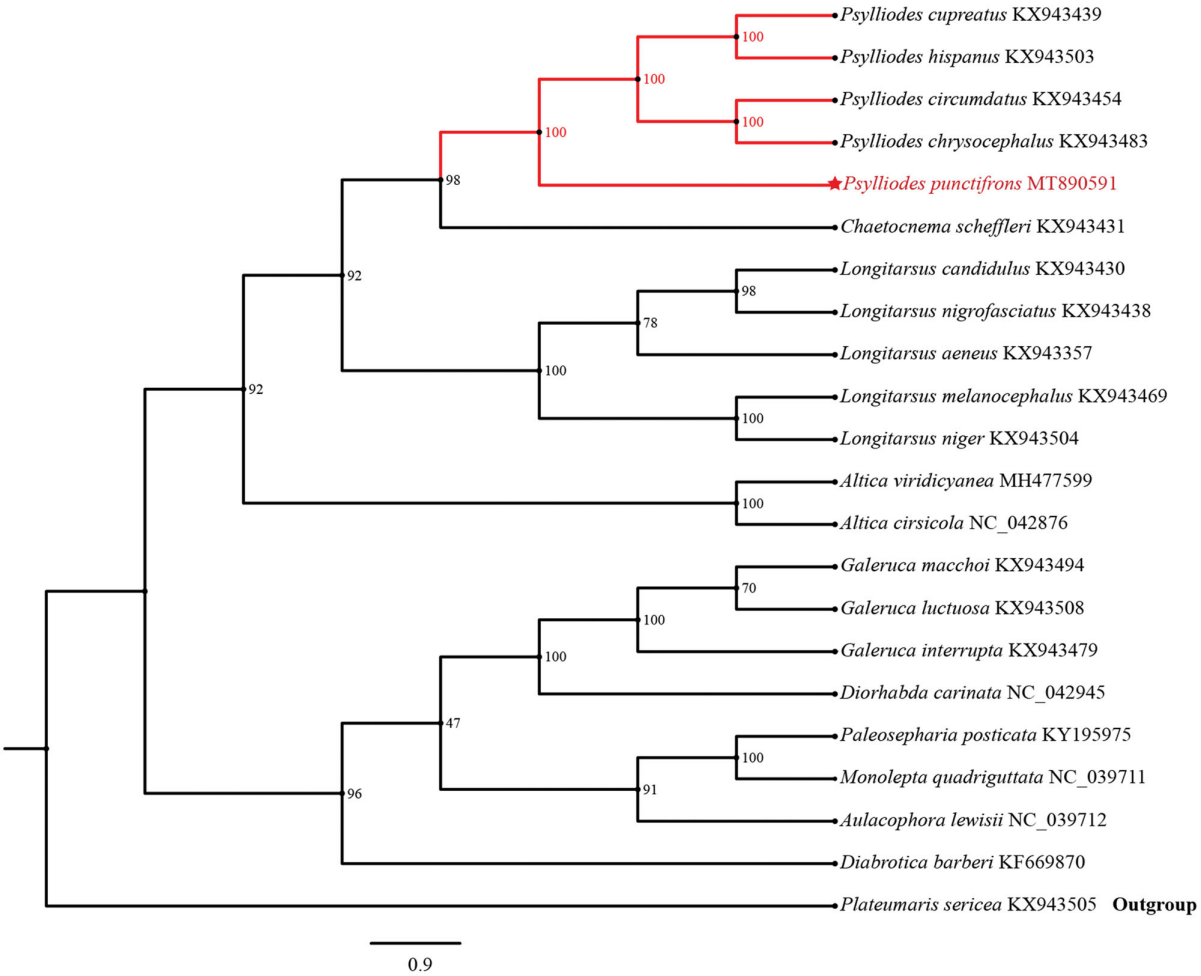
Phylogenetic analyses of *Psylliodes punctifrons* based upon first and second codon positions of the 13 PCGs of 22 species. This analysis was performed using IQ-TREE software. Numbers at nodes are bootstrap values. The accession number for each species is indicated after the scientific name.

## Data Availability

The data that support the findings of this study are openly available in GenBank of NCBI at https://www.ncbi.nlm.nih.gov, reference number MT890591.
